# Comparison of the efficacy between topical diquafosol and artificial tears in the treatment of dry eye following cataract surgery: A meta-analysis of randomized controlled trials

**DOI:** 10.1097/MD.0000000000008174

**Published:** 2017-09-29

**Authors:** Xinyu Zhao, Song Xia, Youxin Chen

**Affiliations:** Department of Ophthalmology, Peking Union Medical College Hospital, Chinese Academy of Medical Sciences, Beijing, China.

**Keywords:** cataract, diquafosol, dry eye, meta-analysis

## Abstract

**Background:**

The prevalence of dry eye following cataract surgery was reported as high as 55.7%, this acute and iatrogenic disorder urgently required appropriate clinical management. The purpose of this study is to compare the efficacy of diquafosol sodium ophthalmic solution (DQS) and conventional artificial tears (AT) for the treatment of dry eye following cataract surgery.

**Methods:**

The PubMed, Embase, and the Cochrane Central Register of Controlled Trials were searched from their earliest entries through June 2017 to obtain the studies, which evaluated the efficacy of DQS for patients with dry eye after cataract surgery. The relevant data were analyzed using StataSE 12.0 software. The PRISMA checklist was used as protocol of the meta-analysis and the guideline was followed. The weighted mean difference, relative risk, and their 95% confidence interval were used to assess the strength of the association.

**Results:**

The authors identified 21 references of which 4 studies evaluating the efficacy of DQS for patients with dry eye after cataract surgery were included. The dataset consisted of 291 patients of dry eye following cataract surgery (371 postoperative eyes). The pooling result of our study suggested that the DQS could significantly better improve the indices like corneal and conjunctival fluorescein staining scores, tear breakup time, and Schirmer I test than AT (*P* < .05). Although the scores of symptom questionnaire could not be pooled, the results of each study also proved that DQS could significantly better relieve the symptoms of postoperative dry eye.

**Conclusion:**

Based on the available evidence, topical DQS has a superior efficacy than AT in the management of dry eye after cataract surgery; however, further researches with larger sample sizes and focus on indicators such as higher-order aberrations, symptom questionnaire scores, and cost-effective ratio are required to reach a firmer conclusion.

## 1 Introduction

With the significant process in development and refining of the surgical techniques and apparatus, today, cataract surgery has become one of the safest and most effective ophthalmic surgical procedures, which are performed on numerous patients. However, many patients still complain of some postoperative symptoms, such as ocular soreness, pain, burning sensation, foreign-body sensation, and poor vision. Until now, effective interventions of patients with those symptoms have not been established.^[1,2]^ In this epoch of patients’ high expectations and premium intraocular lenses, those postoperative discomforts are unacceptable to most patients. According to the results of recent studies, such subjective symptoms are caused by the surgery-induced dry eye.^[3–5]^

As a multifactorial disease of the tears and ocular surface, dry eye is always manifested as ocular discomfort, visual disturbance, and tear film instability, which could cause potential damage to the ocular surface.^[6]^ Several risk factors for postoperative dry eye were reported by previous studies, including forceful opening of the eye lids, thermal damage from the operative microscope, sterilizing conjunctival sac and lids with povidone–iodine, topical anesthetics, transection of the corneal nerves by clear corneal incision, preservatives in eye drops, and topical nonsteroidal anti-inflammatory drugs.^[7–12]^ These factors cause the malfunction of ocular surface epithelium and tear film, then induce dry eye via the vicious cycle between unstable tear film and damaged epithelium.^[4,13,14]^ According to the Japanese criteria of dry eye, the prevalence of postoperative dry eye was reported to be as high as 55.7%, this acute and iatrogenic disorder urgently required appropriate clinical management.^[4,13–15]^

With deeper understanding of the pathophysiology of dry eye, the optimal intervention has shifted from simply lubricating the ocular surface by artificial tears (AT) or sodium hyaluronate to applying medications to increase the secretion of water and mucin. Recently, 3% diquafosol sodium ophthalmic solution (DQS) was approved for the treatment of dry eye. DQS is a purinergic P2Y2 receptor agonist, which may stimulate the secretion of water and mucin from conjunctival epithelial cells and goblet cells.^[16,17]^ Previous studies reported that DQS could effectively relieved various symptoms of dry eye and improve the visual function of the dry eye patients.^[17–19]^

However, the effect of DQS on dry eye after cataract surgery was scarcely studied and relatively unclear, the sample sizes of previous studies were relatively small and may not detect the difference as statistically significant.^[15,20–22]^ In addition, some studies suggested that the addition of DQS or AT may equally relieve dry eye after cataract surgery.^[22]^ As DQS is significantly more expensive than common AT, we need more solid evidence to conclude whether the application of DQS in our routine regimen of postoperative therapy for dry eye following cataract surgery is appropriate.

Until now, no meta-analysis in this field has focused on this problem. Thus, we undertook a meta-analysis to evaluate the efficacy of DQS for the treatment of dry eye following cataract surgery, in order to provide a reference for the decision making of ophthalmologists.

## 2 Materials and methods

This meta-analysis was performed strictly according to the guidelines, the “preferred reporting items for systematic reviews and meta-analysis (the PRISMA statement).”^[23]^ Since this is a systematic review, ethical approval is not required.

### 2.1 Search strategy

The PubMed, Embase, and the Cochrane Central Register of Controlled Trials were searched from their earliest entries through June 2017. The following keywords or corresponding Medical Subject Headings (Mesh) were used: “cataract surgery”, “diquafosol”, and “Randomized Controlled Trial”. The detailed electronic search strategy of PubMed was ((((“Randomized Controlled Trial” [Publication Type]) OR random ∗ [Title/Abstract])) AND ((diquafosol [Title/Abstract]) OR “diquafosol” [Supplementary Concept])) AND (((“Cataract Extraction” [Mesh] OR “Cataract” [Mesh])) OR Cataract [Title/Abstract]). The searches started at May 25, 2017 and ended at June 1, 2017. The reference lists of the relevant articles were also manually reviewed to further identify potentially related studies. No language restriction was imposed.

### 2.2 Inclusion criteria and exclusion criteria

Inclusion criteria were participants: patients with visually significant cataract; intervention: cataract surgery; comparison: postoperative dry eye managed with the use of diquafosol versus AT; outcomes: at least one of the followings: tear breakup time (BUT), corneal and conjunctival fluorescein staining scores, subjective symptom questionnaire, and Schirmer I test; and methodological criterion: randomized controlled trial.

Exclusion criteria were other differences between the experimental group and control group beside the administration of diquafosol and AT; relative risk (RR) or weighted mean difference (WMD) could not be estimated as insufficient data; animal studies or cadaver subjects; and redundant publications.

### 2.3 Data extraction and assessment of methodological quality

After obtaining the list of potential relevant articles, the Endnote software was used to remove the duplicates. Then the titles and abstracts of the remaining studies were reviewed to filter out the unrelated articles. The next procedure was achieving the full text of each article and reviewing them, the studies that met the eligibility criteria and fail the exclusion criteria were included. Two authors (XZ and SX) extracted relevant data independently, including the first author's name, publication year, design, sample size (patients and eyes), group size, average age, gender ratio, application method, other intervention protocol, and outcome. The corresponding authors of the included articles would be contacted if the requisite data were unavailable. The data of updated publications involving same cohort of cases would be extracted synthetically. Two authors (YZ and SX) independently assessed the methodological quality of each included randomized controlled trial by 12-item scale^[24]^: a trial with a score of 7 or more was considered high quality, more than 4 but no more than 7 was considered moderate quality, and no more than 4 was considered low quality. Disagreements were evaluated by kappa text and were resolved by discussing with the corresponding author (Y-XC).

### 2.4 Statistical methods

Statistical analyses were performed with StataSE 12.0 software (StataCorp, College Station, TX). The WMD and 95% confidence interval (CI) were calculated for continuous data, and the RR and 95% CI were calculated for dichotomous data. Chi-squared test and I^2^ were used to evaluate the statistical heterogeneity. If heterogeneity was low (*P* > .1, I^2^ < 50%), a fixed-effect model would be used. If substantial heterogeneity existed (*P* < .1, I^2^ > 50%), both sensitivity analysis and subgroup analyses would be performed to identify the source of the heterogeneity. If the heterogeneity could not be eliminated, a random-effect model would be used when the result of meta-analysis had clinical homogeneity, or a descriptive analysis would be used.

Publication bias was evaluated by Begg funnel plot and the Egger linear regression test, a *P* < .05 was considered to indicate statistical significance.^[25]^

## 3 Results

### 3.1 Study characteristics

**Figure 1:**
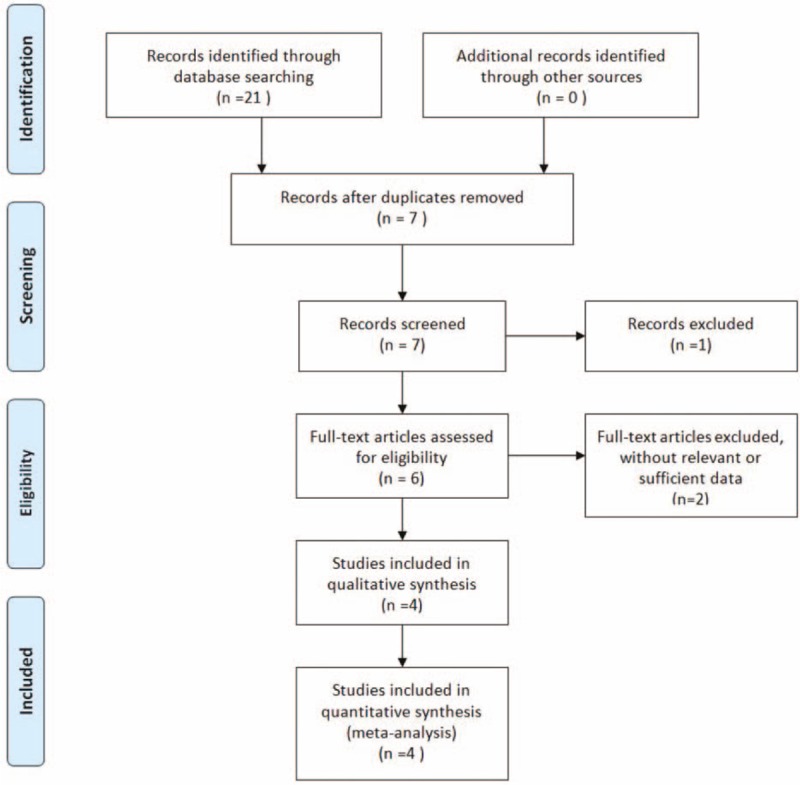
Flow chart summarizing the selection process.

Initially, 21 potentially relevant studies were identified in our search. We firstly removed 14 duplicate studies by Endnote software, then screened off 1 unrelated article after reviewing titles and abstracts, and excluded 2 studies for their unrelated data after reviewing the full texts. Eventually, 4 studies were included in our meta-analysis. The dataset consisted of 291 patients of dry eye following cataract surgery, including 371 postoperative eyes. Among them, 178 eyes were in the DQS group and 193 eyes in the AT group. The sample sizes varied from 32 to 154 patients. The demographic characteristics of the case group and control group were similar in every study. Table 1 described the main characteristics of the included studies. Figure 1 shows the literature-exclusion procedures. The 12-item scale was used to assess the methodological quality of the included studies (Table 2), the average score was 10.00 ± 0.82 and all the included studies were of high quality. Excellent inter-rater agreement (κ = 0.76) was achieved between the investigators regarding eligibility.

**Table 1:**
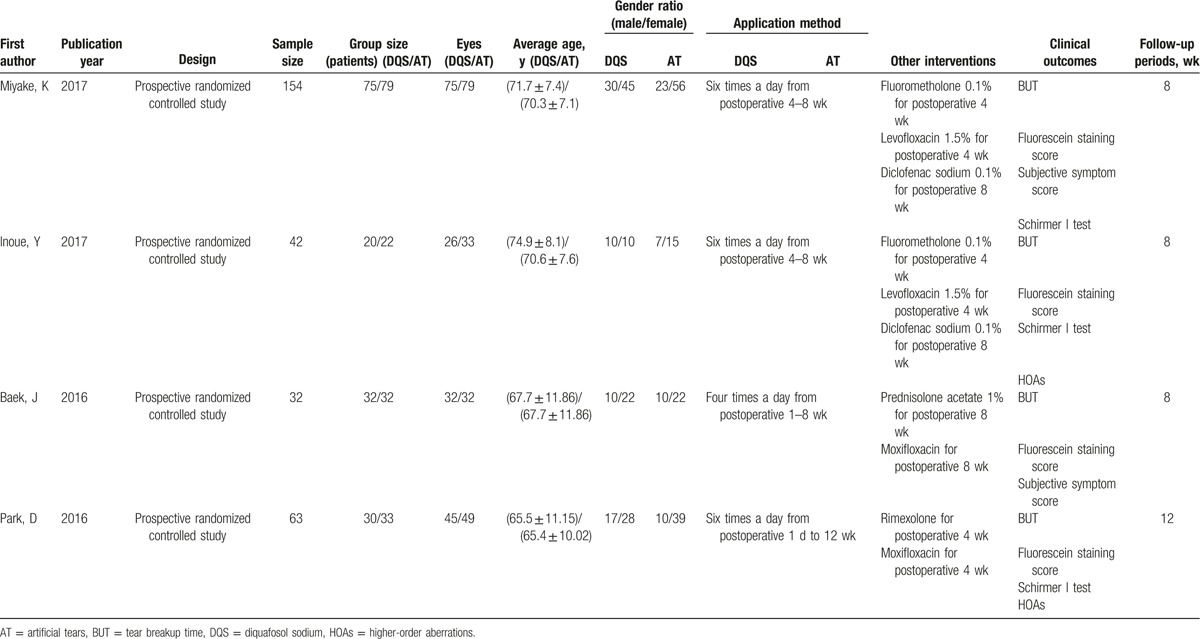
Main characteristics of the included studies.

**Table 2:**
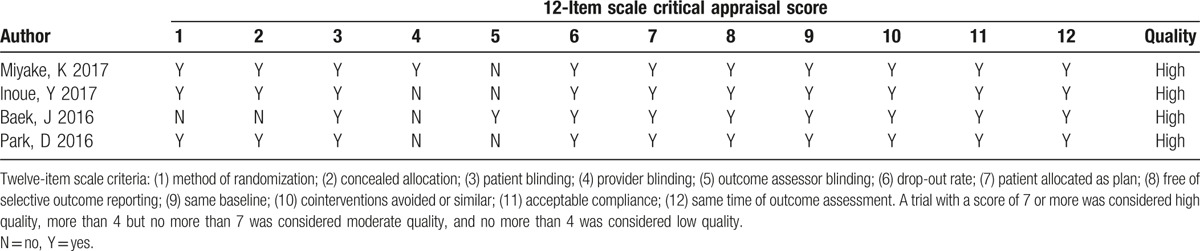
Twelve-item scale critical appraisal scores.

### 3.2 Tear breakup time

**Figure 2:**
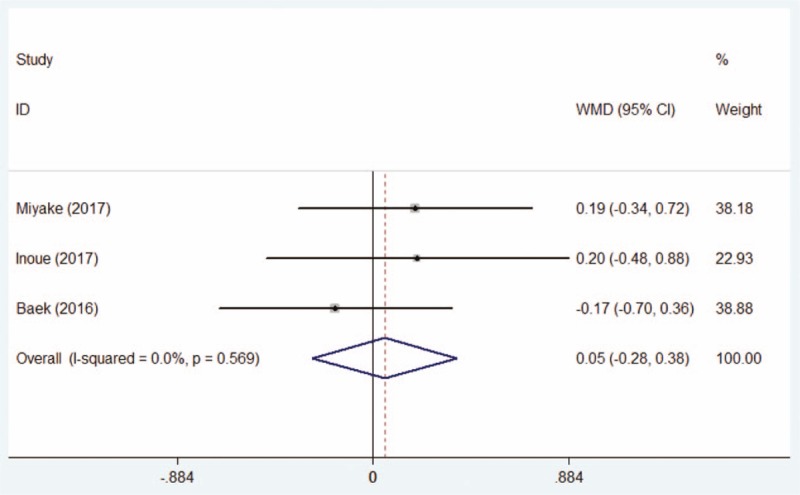
Comparison of pretherapeutic tear break time between the DQS group and AT group. AT = artificial tears, DQS = diquafosol sodium ophthalmic solution.

Together, 3 studies^[15,20,21]^ included 133 eyes in the DQS group and 144 eyes in the AT group described the BUT before the treatment of postoperative dry eye. When pooling the data of pretherapeutic BUT, fixed-effect models were used as Chi-squared test manifested no heterogeneity. The results of meta-analysis showed no statistical difference of BUT between the 2 groups before the treatment (WMD = 0.05, 95% CI: −0.28 to +0.38, Fig. 2).

**Figure 3:**
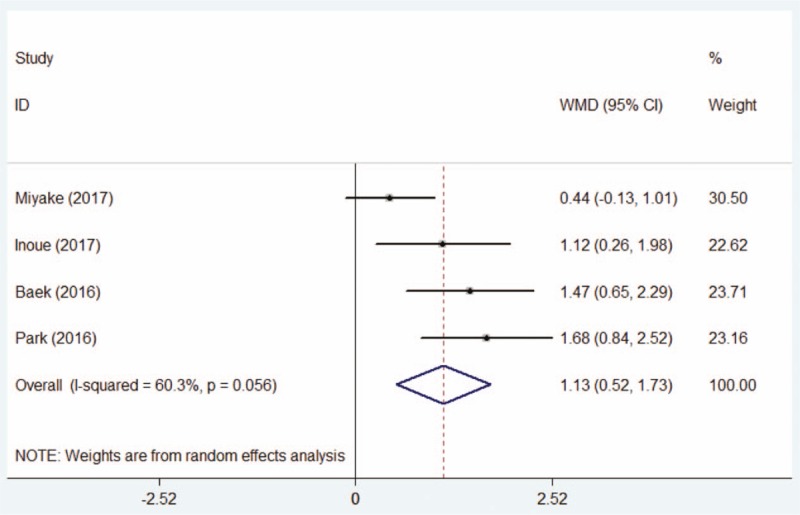
Comparison of post-therapeutic tear break time between the DQS group and AT group. AT = artificial tears, DQS = diquafosol sodium ophthalmic solution.

Four papers^[15,20–22]^ described the post-therapeutic BUT, including 178 eyes in DQS group and 193 patients in AT group. Sensitivity analysis and subgroup analysis failed to eliminate the detected heterogeneity (*P* = .056, I^2^ = 60.3%). The random-effect model was used for the reason that regardless of the exclusion or inclusion of every study, the pooling results were all same and all studies were of high quality. The forest plot indicated that the post-therapeutic BUT of DQS group was significantly increased than that of AT group (WMD = 1.13, 95% CI: 0.52–1.73, Fig. 3).

### 3.3 Schirmer I test

**Figure 4:**
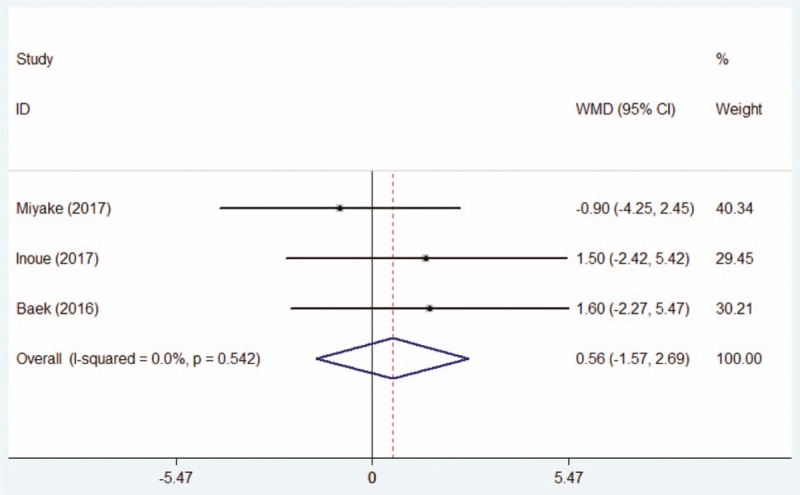
Comparison of pretherapeutic Schirmer I test between the DQS group and AT group. AT = artificial tears, DQS = diquafosol sodium ophthalmic solution.

The pretherapeutic results of Schirmer I test for patients with postoperative dry eye were reported in 3 studies.^[15,20,21]^ Fixed-effect models were used as no heterogeneity was detected. The forest plot manifested that there was no statistical difference between the DQS group and the AT group (WMD = 0.56, 95% CI: −1.57 to +2.69, Fig. 4).

**Figure 5:**
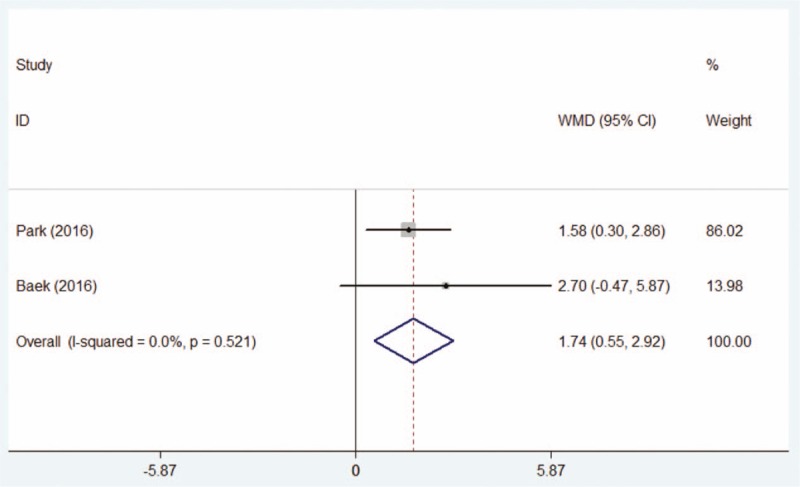
Comparison of post-therapeutical Schirmer I test between the DQS group and AT group. AT = artificial tears, DQS = diquafosol sodium ophthalmic solution.

Two studies^[21,22]^ calculated the post-therapeutic results of Schirmer I test, the pooling result by fixed-effect model manifested that the results of Schirmer I test of the DQS group was significantly increased than those of AT group (WMD = 1.74, 95% CI: 0.55–2.92, Fig. 5).

### 3.4 Corneal and conjunctival fluorescein staining scores

**Figure 6:**
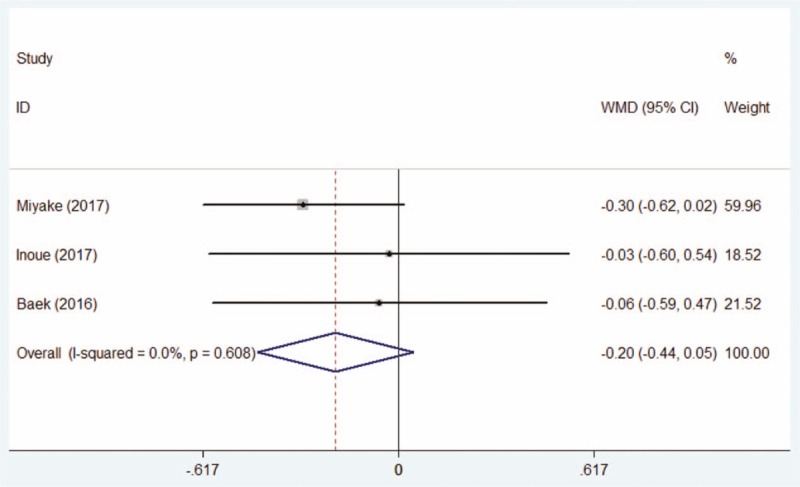
Comparison of pretherapeutic corneal and conjunctival fluorescein staining scores between the DQS group and AT group. AT = artificial tears, DQS = diquafosol sodium ophthalmic solution.

Totally, 3 studies^[15,20,21]^ included 133 eyes in the DQS group and 144 eyes in the AT group described the pretherapeutic corneal and conjunctival fluorescein staining scores for patients with postoperative dry eye. The meta-analysis by fixed-effect model indicated that there was no statistical difference between the DQS group and the AT group (WMD = −0.20, 95% CI: −0.44 to +0.05, Fig. 6).

**Figure 7:**
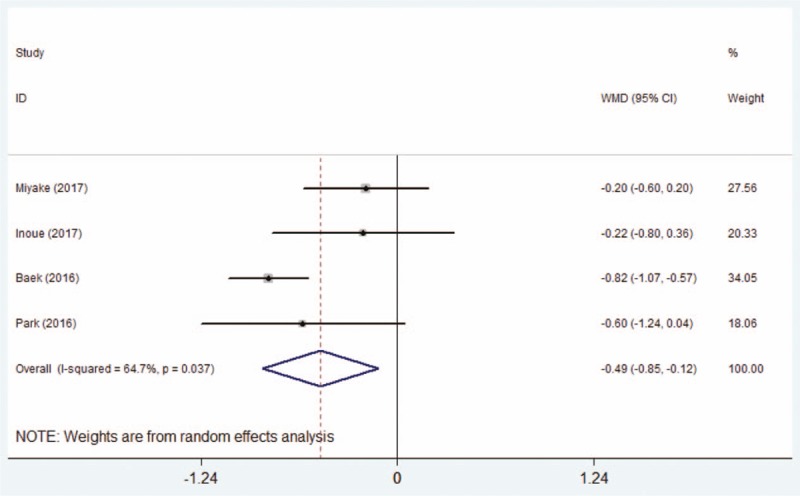
Comparison of post-therapeutic corneal and conjunctival fluorescein staining scores between the DQS group and AT group. AT = artificial tears, DQS = diquafosol sodium ophthalmic solution.

The post-therapeutic corneal and conjunctival fluorescein staining scores were calculated in all 4 studies.^[15,20–22]^ The pooling results by random-effect model manifested that the corneal and conjunctival fluorescein staining scores of the DQS group was significantly lower than that of AT group (WMD = −0.49, 95% CI: −0.85 to −0.12, Fig. 7).

### 3.5 Symptom questionnaire scores

Miyake and Yokoi^[20]^ and Baek et al^[21]^ evaluated the pre- and post-therapeutic symptom questionnaire scores between the DQS group and AT group, their results manifested that DQS could significantly better relieve the symptoms in the questionnaire than AT. However, the data could not be pooled as different questionnaires were used.

### 3.6 Publication bias

Begg test (*P* = 1.00, continuity corrected) and Egger test (*P* = .338) indicated that publication bias did not affect our results.

## 4 Discussion

By exhibiting purinoceptor P2Y_2_ receptor agonist activity, DQS cannot only promote the secretion of water and secretory mucin from conjunctival tissue, but also increase the expression of membrane-associated mucin, these 2 mechanisms could improve tear volume and quality.^[26,27]^ Mucin is extensively distributed on the surface of mucous tissues to moisten them and prevent them from damage. The shortage of mucin on ocular surface reduced its wettability and induced unstable tear film, those are regarded as the pathogenesis of short BUT-type dry eye.^[28–30]^ The risk factors for postoperative dry eye, such as light and heat from the operative microscope, vigorous intraoperative irrigation of the tear film, preservatives in eye drops, and cleaning of the conjunctival sac and lids with povidone–iodine, etc., damage the ocular surface epithelium and the tear film and then induce dry eye via the vicious cycle between them.^[7–12]^ Therefore, the dry eye following cataract surgery is a nonphysiological iatrogenic and acute disorder. By using DQS, we can manage this disorder with a shift from simply lubricating and hydrating the ocular surface with products, such as AT, to more reasonable methods of enhance the secretion of water and mucin.

By evaluating the indices such as corneal and conjunctival fluorescein staining scores, BUT, and Schirmer I test, our study suggested that the DQS is more beneficial than AT in the treatment of dry eye following cataract surgery. Although the scores of symptom questionnaire could not be pooled, the results of each study also proved that DQS could significantly better relieve the symptoms of postoperative dry eye.

Previous studies reported the close relationship of dry eye with higher-order aberrations (HOAs). Unstable tear film might increase HOAs and contribute to glare, halo, decreased night vision, and impaired contrast sensitivity.^[31,32]^ For dry eye patients following cataract surgery, optimal visual outcomes could be achieved if DQS could reduce HOAs; however, our study could not evaluate this index as insufficient data.

As far as we know, this is the first meta-analysis evaluating the efficacy of DQS and AT for treatment of dry eye following cataract surgery that includes all the available evidence of high quality. The satisfactory heterogeneity and the insignificant publication bias made our study highly reliable and might provide valuable instructions for ophthalmologist. However, the following limitations still exist: although all the available data had been pooled together by the most reliable way, the final sample size in our meta-analysis was still relatively small, more researches of high quality were need to get a more solid conclusion; a descriptive analysis was used for symptom questionnaire scores; and there were insufficient data to analyze the HOAs. Further researches should focus on these 2 points as they are the most direct indices for evaluating the efficacy of 2 drugs; currently, DQS is only approved in Japan and South Korea, the possible effect of ethnic differences should be further evaluated; as DQS is significantly more expensive than common AT, cost-effective ratio should also be evaluated to get a more solid evidence to conclude whether the application of DQS in our routine regimen of postoperative therapy for cataract surgery is appropriate.

## 5 Conclusions

Based on the available evidence, DQS has a superior efficacy than AT in the management of dry eye after cataract surgery, supported by better corneal and conjunctival fluorescein staining scores, BUT, and Schirmer I test. Given the limitations in our study, further researches with larger sample sizes and focus on indicators such as HOAs, symptom questionnaire scores, and cost-effective ratio are required to reach a firmer conclusion.

## Acknowledgments

The authors would like to thank the editor and anonymous reviewers for the valuable comments and suggestions.
